# Treating Posttraumatic Stress Disorder in Female Victims of Trafficking Using Narrative Exposure Therapy: A Retrospective Audit

**DOI:** 10.3389/fpsyt.2017.00063

**Published:** 2017-06-01

**Authors:** Katy Robjant, Jackie Roberts, Cornelius Katona

**Affiliations:** ^1^Clinical Department, Helen Bamber Foundation, London, United Kingdom; ^2^Duke Street Practice, London, United Kingdom; ^3^Division of Psychiatry, University College London, London, United Kingdom

**Keywords:** human trafficking, modern slavery, narrative exposure therapy, posttraumatic stress disorder, sexual- and gender-based violence

## Abstract

**Background:**

Human trafficking is a form of modern slavery that involves the forced movement of people internally within countries, or externally across borders. Victims who are trafficked for sexual exploitation are subject to repeated, multiple trauma, and high rates of mental health problems including posttraumatic stress disorder (PTSD) have been found. Narrative exposure therapy (NET) is an evidence-based treatment for PTSD.

**Methods:**

In this retrospective audit, we record the results of NET to treat 10 women who had been trafficked for sexual exploitation who were diagnosed with PTSD.

**Results:**

All 10 women completed the therapy and experienced a reduction in PTSD severity scores at posttreatment, with improvements that were maintained or further improved at 3-month follow-up. General distress was also significantly reduced following treatment.

**Conclusion:**

Although limited by sample size and retrospective design, this audit demonstrates that NET is a feasible treatment for PTSD in this population and warrants further evaluation in a randomized controlled trial. Further adjunctive interventions may also be necessary to treat the additional psychological problems experienced by this population.

## Introduction

Human trafficking is a form of modern slavery that involves the forced movement of people internally within countries, or externally across borders. The UN Trafficking Protocol (1) defines human trafficking as “the recruitment, transportation, transfer, harboring or receipt of persons, by means of the threat or use of force or other forms of coercion, of abduction, of fraud, of deception, of the abuse of power or of a position of vulnerability or of the giving or receiving of payments or benefits to achieve the consent of a person having control over another person, for the purpose of exploitation.”

Individuals can be trafficked for a wide range of purposes including forced labor, domestic servitude, petty crime, cannabis farming, and sexual exploitation (2). People who have been trafficked have frequently encountered extreme violence and psychological abuse during their enslavement (3).

Although in its infancy, research into the mental health impact of trafficking has consistently found high rates of mental health problems, most commonly depression, anxiety, and posttraumatic stress disorder (PTSD) (4–8). A number of factors are likely to contribute to this, including the interplay between genetic predisposition and environmental factors such as pre-migration experiences, experiences within the trafficking situation, and the post-release context, including access (or otherwise) to therapeutic interventions.

In a study of trafficked women returning to Moldova (4), several risk factors for mental disorders were identified, including childhood sexual abuse, unmet needs, and lack of social support post-trafficking. Exposure to violence prior to the trafficking experience was also linked with PTSD, depression, self-harm and anxiety in trafficked children, and adolescents attending post-trafficking services in Thailand, Cambodia, and Vietnam (9).

Experiences that occur within the trafficking situation also contribute to subsequent mental health problems. Longer periods within the trafficking situation in which they were sexually exploited are associated with greater post-trafficking mental health problems among trafficked women returning to Moldova (4) and among survivors of trafficking for sexual exploitation in the USA (6). Similarly, women who were trafficked for at least 6 months were twice as likely to experience higher levels of anxiety or depression (10). This may be explained by increased exposure to violence or sexual exploitation over time. Some studies have shown a specific relationship between events that occur during the trafficking experience and mental health problems. For example, sexually exploited women in Nepal were shown to have higher rates of anxiety, depression, and PTSD than those who were victims of non-sexual exploitation (11). In a study assessing mental health problems in women accessing post-trafficking services in a range of countries, sustaining physical injuries and sexual violence were linked to higher rates of depression, anxiety, and PTSD (10). In a study of 1,102 trafficked men, women, and children, several factors (threats, severe violence, poor living conditions, long working hours, and unfair loss of pay) were linked to increased likelihood of developing symptoms of depression, anxiety, and PTSD (5). In addition to the type of trauma experienced within the trafficking situation, the individuals’ psychological experience during the trafficking situation is also likely to be important. Survivors who had the most marked subjective feelings that their freedom was restricted while they were being trafficked had increased rates of anxiety (10) and double the overall risk of poor mental health (5) than those who felt less restricted.

Although mental health problems among victims of trafficking have been shown to be high, recovery without treatment is rare, particularly in those who have developed PTSD (10). Where there is comorbidity, recovery often does not occur even when rehabilitation has been attempted (7). This is unsurprising given the multiplicity of trauma that victims of trafficking have experienced (2), which often includes trauma prior to the trafficking situation. In keeping with this, there is evidence from the wider (non-trafficking) literature that PTSD is more common among those who have experienced multiple trauma (12) and recovery is less likely (13).

There is a paucity of research into clinical interventions for the treatment of victims of trafficking. Much of the literature in this area proposes principles by which interventions should be delivered rather than describing treatment trials.

Such trials are in any case challenging because of the many other adversities that survivors of trafficking often also experience. Those who have been trafficked across borders face immigration insecurity. Many seek asylum in the same country in which they have escaped their captors. This may increase their fears about the possibility of being recaptured. Victims of trafficking may also experience significant stressors related to their insecure immigration status such as further incidents of imprisonment (for example, for having used false documents while under the control of traffickers), or of immigration detention. Research has consistently demonstrated that a long asylum determination procedure and other post-migration adversities contribute to mental health problems (14–16). Immigration detention has also been shown to be associated with mental health problems (17).

Given the high rate of PTSD among victims of trafficking, an evidence-based treatment for PTSD should be available and be provided to those victims who meet diagnostic criteria for this condition. Narrative exposure therapy (NET) (18) is a short-term evidence-based treatment for PTSD that was specifically developed for victims of multiple trauma and may therefore be worthy of evaluation in the context of trafficking-related PTSD.

In NET, an individual is taken through their entire autobiography. Both traumatic and positive events are identified and understood within the wider sociopolitical context in which they occurred. The majority of time in therapy is spent carrying out a detailed exploration of, and exposure to, traumatic events experienced chronologically and in context. The nature of the exposure to traumatic events in NET is different to reliving in CBT and also to other forms of therapies for PTSD. The therapist guides the exposure through the event in a much more directive manner than is usual in other therapies. Very close attention is paid to the emotional and physiological and behavioral responses that the client experiences during the exposure, and these are reflected back to guide the client through the retelling of the trauma at the same time as maintaining a dual focus on present and past experiences in a natural manner that resembles an “exposure conversation.” Physical experiences occurring both peritraumatically and during the exposure are attended to with as much emphasis as cognitions, meanings, emotions, and sensory experiences. Unlike some other trauma treatments, dissociation and affect regulation are managed and treated while the exposure is occurring, rather than in a preliminary “stabilization” phase of treatment. Contextual information is integrated into the memory for the trauma during the exposure, in order that the autobiographical memory is completed. The detailed account of the autobiography is transcribed by the therapist and is given to the client at the end of therapy. It provides a written acknowledgment or testimony of their experiences, including both traumatic events and positive events, within the context in which they occurred. The therapist “bears witness” to the client’s story and actively facilitates the client to speak in detail about the traumatic events in the context of the overall narrative of their life story.

Narrative exposure therapy was initially developed for use in low income countries in contexts of ongoing insecurity and high likelihood of exposure to further trauma. Subsequently, it has been shown also to be effective among asylum seekers and refugees in high income countries. Several trials have now demonstrated its efficacy among diverse populations, including former child soldiers (19), asylum seekers in Germany who had experienced war and torture (20), and individuals with comorbid BPD (21). These populations have in common a high prevalence of PTSD resulting from experience of multiple traumas, which in some cases includes developmental trauma.

In the light of the above, and particularly because of the multiple traumas to which victims of human trafficking have usually been subjected, NET was therefore chosen in this pilot study as an appropriate treatment for women trafficked for sexual exploitation who suffered from PTSD. Victims of trafficking for sexual exploitation are likely to experience high levels of shame. In NET, shame is considered to be one of several emotions that are likely to occur during sexual violence including rape. Shameful events are treated in the same way as any other traumatic event. The close attention and empathic response of the therapist is thought to allow the individual feeling shame to connect with another compassionate person as they go through the exposure to this event. Rather than experiencing the response that they expect (being rejected and socially excluded), the client instead experiences empathy and compassion. The rapid interplay between therapist and client during exposure in NET allows the client to feel that they are not alone and to experience the therapist’s acceptance of the client. In NET, the therapist is not neutral, but rather is an advocate for the Human Rights of survivors (22). The therapist facilitates the expression of emotions that the survivor was not able to experience and express at the time. In our collective clinical experience, this often enables the client to take a position of acknowledging the abuse of their Human Rights. Anger is a powerful antidote to shame.

Many victims of trafficking have had their trust betrayed by traffickers who may have initially engaged in romantic relationships with their victim, prior to exploiting them. In our clinical experience victims of trafficking often continue to hold ambivalent feelings toward their trafficker and may develop new relationships with abusive partners, or experience further exploitation by others. In order to help the victim to recognize and acknowledge the exploitation that they have been subjected to by the person who trafficked them, and to attempt to increase the interpersonal safety of the victim in the present and future, an emphasis on attending to the possible motivations of the traffickers was included during the NET sessions. Given the potential risk of further exploitation, a “safety review” was performed at the beginning of each session, in which individuals were asked about new individuals that they had met over the course of the week and whether the client or their families had had any contact with traffickers or their associates.

## Materials and Methods

### Participants

The participants were clients of the Helen Bamber Foundation, a charity that provides specialist psychological therapy as part of a holistic model of care to victims of trafficking among others. Ten female victims of trafficking for sexual exploitation were treated using NET. All clients of the Helen Bamber Foundation who are offered therapy are asked to complete the measures described below as a means of evaluating outcome. All female victims of trafficking who meet diagnostic criteria for PTSD and who live in London where the center is based are offered psychological therapy.

The case series described represents a retrospective audit of the first 10 female victims of trafficking who received treatment with NET. All of the participants were provided with information about the therapy and associated risks and benefits. All provided written consent to receiving treatment. Those who declined treatment with NET were offered alternative interventions and could still access the other services offered by the Helen Bamber Foundation. Nine out of the 10 participants had insecure immigration status at the time of starting treatment; 5 of the women were from Albania, 4 were from different African countries, and 1 woman was from China. The women were aged between 18 and 48. All had experienced multiple traumatic events during childhood and in the context of the trafficking situation. All had been subjected to multiple sexual assaults as well as severe physical abuse in the trafficking situation. Six out of 10 were treated with the help of professional interpreters.

### Instruments

The Posttraumatic Diagnostic Scale (PDS) (23) was used to assess and diagnose PTSD. Where necessary, the PDS interviews were conducted with the help of an interpreter. Since language and literacy issues meant that participants were not able to complete the questionnaires themselves, the therapist administered the PDS as an interview by reading the questionnaire to the participant. All 10 participants were identified as having PTSD according to the PDS. The Clinical Outcomes in Routine Evaluation (CORE), a self-report 34-item rating scale was used as a measure of overall distress (24). Where necessary, participants completed the CORE with the help of an interpreter.

### Procedure

All clients provided consent to participate in both the diagnostic interviews and the treatment. Following the initial diagnostic interview, all were offered NET. They received between 10 and 19 sessions (mean = 14.3, SD = 3.92). They completed the same measures at the end of treatment and at a 3-month follow-up. Treatment fidelity was maximized through close case supervision by the first author, who has considerable experience in delivering NET and training others to apply the approach.

## Results

All clients completed 10–19 sessions of treatment, depending on the number of traumatic events that they had experienced. Prior to treatment, all clients were in the severe range for PTSD, according to the PDS (mean = 43, SD = 2.91 range = 37–47). At end of treatment, scores ranged from mild to moderate to severe (mean = 14.2, SD = 6.71) At 3-month follow-up, scores ranged from mild to moderate (mean = 10.6, SD = 5.2).

### Posttraumatic Diagnostic Scale

**Figure 1:**
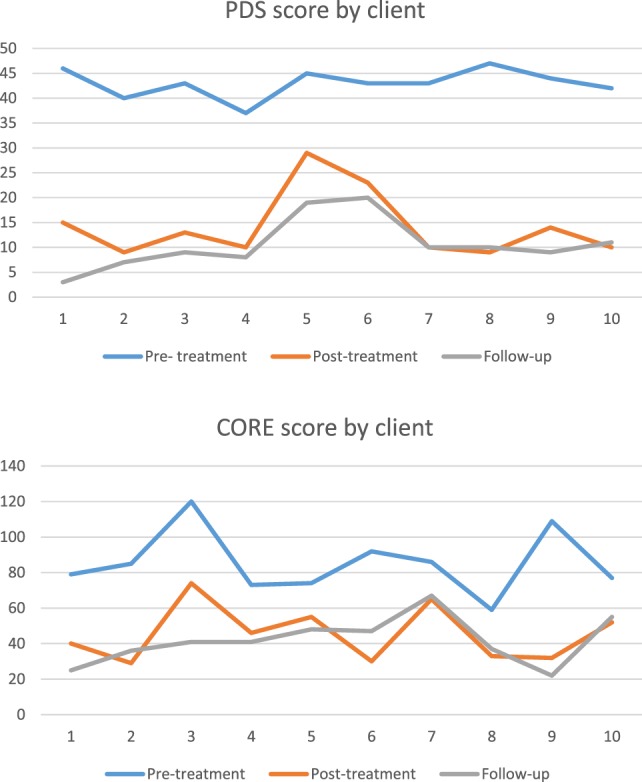
**PDS and CORE scores by client**.

A one-way repeated measures ANOVA was conducted to assess the effect of psychotherapy on PDS [assumed sphericity *d*: Mauchly’s *W*(2) = 0.833, *p* = 0.482]. There was a significant effect of psychotherapy on PDS [*F*(2,18) = 205.075, *p* < 0.001, partial η^2^ = 0.958, *n* = 10] (Figure 1). Three, Bonferroni-corrected, paired samples *t*-tests were conducted for *post hoc* comparisons between conditions. There was a significant difference between PDS scores before therapy (M = 43.0, SD = 2.91) and those directly after (M = 14.2, SD = 6.71, *p* < 0.001) and 3 months after psychotherapy (M = 10.6, SD = 5.19, *p* < 0.001). PDS scores directly and 3 months after psychotherapy did not significantly differ (*p* = 0.088). The effect size of *f* = 4.78 indicates a strong effect of therapy on PDS scores.

### Clinical Outcomes in Routine Evaluation

A one-way repeated measures ANOVA was conducted to assess the effect of psychotherapy on CORE [assumed sphericity: Mauchly’s *W*(2) = 0.660, *p* = 0.189]. There was a significant effect of psychotherapy on CORE scores [*F*(2,18) = 29.874, *p* < 0.001, partial η^2^ = 0.768, *n* = 10]. Three Bonferroni-corrected, paired samples *t*-tests were conducted for *post hoc* comparisons between conditions. Results indicated that there was a significant difference between CORE scores before psychotherapy (M = 85.4, SD = 17.92) and those directly after (M = 45.6, SD = 15.66, *p* < 0.001) and 3 months after psychotherapy (M = 41.9, SD = 13.36, *p* = 0.001). CORE scores directly and 3 months after psychotherapy did not significantly differ (*p* = 1.000). The effect size of *f* = 0.73 indicates a strong effect of therapy on CORE scores.

## Discussion

The NET case series demonstrated a substantial and clinically significant reduction in PTSD symptoms and in emotional distress between the beginning and the end of NET treatment, which remained statistically significant at 3-month follow-up. Although the intervention was therefore effective overall, three clients still had PTSD symptoms in the moderate range at follow-up. However, all participants had scored within the severe range prior to treatment. None of the consecutive series of clients identified dropped out of treatment. This suggests that NET is tolerated and acceptable to victims of trafficking for sexual exploitation, even though all of the participants had experienced events that are likely to increase shame and that shame [a clinically important aspect of PTSD that is now formally recognized within the “negative alterations in cognition and mood” criterion in the current edition of the Diagnostic and Statistical Manual of Mental Disorders (DSM 5) (25)] was regularly reported through the exposure sessions.

Although limited by sample size and design, the results are useful given the paucity of existing evidence about the benefits of treatment in victims of trafficking. Without a control group the findings cannot be interpreted to show that NET is effective for this population, and an RCT would be required to explore this. It is also problematic that the participants completed the PDS *via* an interview with the therapist, potentially introducing bias. A further limitation of the study is that we did not control for the impact of post-migration stressors and also of any significant positive events that may have occurred during the therapy or between the end of therapy and follow-up. For example, some of the clients may have received positive or negative decisions in relation to their immigration status during this time, which may have impacted their levels of general distress, but would be less likely to have influenced PTSD scores.

Despite its limitations, this small pilot demonstrates the potential usefulness for NET as a treatment of PTSD in severely traumatized victims of trafficking for sexual exploitation. This is advantageous since it was developed for use in low income countries and has been shown to be effective when disseminated through the training of unskilled counselors to provide treatments (25). Given the scale of human trafficking, this may therefore be an important requirement when choosing treatment approaches. The addition of the safety review enabled the therapist to monitor risk of further exploitation during treatment, and the attention to processing of the motivations of others appeared to facilitate acknowledgment of past exploitation. This, together with the reduction of PTSD symptoms, may lead to a reduction of future risk of exploitation. The fact that this pilot demonstrated both a reduction in PTSD symptoms and overall distress and was well tolerated by participants, suggests that it warrants further exploration in a randomized controlled trial. Further research is also required to see whether this treatment has an effect on risk of further exploitation.

Not all individuals who have been trafficked for sexual exploitation will develop PTSD. Other difficulties including anxiety and depression have been observed (4–9). In our clinical experience we have also observed a marked lack of agency, and significant difficulties in practicing assertiveness and in making decisions. These may be explained by experiences within the context of the trafficking. Many victims are not able to make any choices about their lives, even in terms of basic functioning. For example, many victims describe being unable to choose when they eat or drink, sleep, or use the toilet. Many had been held in conditions in which they are completely controlled, physically (through the use of locked rooms or restraints) and/or psychologically. Following escape or rescue from such trafficking situations, many women described being unable to make even simple decisions for themselves, and lack basic assertiveness skills. Therefore, additional interventions may be necessary in order to assist them to integrate effectively into life out of captivity.

## Conclusion

Women who have been trafficked for the purpose of sexual exploitation are likely to have experienced multiple traumatic events and are at risk of PTSD. Little is known about effectiveness of treatments for this group. In this pilot study, NET was found to be a well-tolerated treatment that was effective in reducing PTSD scores. Additional psychological problems faced by this group are likely to influence the risk of future exploitation, and additional interventions are necessary to target this.

## Ethics Statement

The study represents audit findings and therefore ethical approval was not applied for in advance. Signed informed consent was obtained from all participants. The consent process took into account the vulnerability of the patient group involved.

## Author Contributions

KR was responsible for conceptualization, delivery of treatments, data collection, data analysis, and drafting of manuscript. JR made significant contributions to treatment delivery, data collection, and manuscript revisions. CK made significant contributions to manuscript revisions. All authors read and approved the final manuscript.

## Conflict of Interest Statement

The authors declare that the research was conducted in the absence of any commercial or financial relationships that could be construed as a potential conflict of interest.
